# Supplementary material for: The Effect of Molecular Weight on the Solubility Properties of Biocompatible Poly(ethylene succinate) Polyester
Source: Polymers (Basel). 2021 Aug 15;13(16):2725. doi: 10.3390/polym13162725 (PMC8398594; doi:10.3390/polym13162725)
Supplement: Supplementary file 1 [file polymers-13-02725-s001.zip › polymers-1325925-supplementary.pdf]

polymers

Supplementary information

The Effect of Molecular Weight on the Solubility Properties of Biocompatible Poly(ethylene succinate) Polyester

Mohamed M. Abdelghafour [1,2], Ágoston Orbán [1], Ágota Deák [1], Łukasz Lamch [3], Éva Frank [4], Roland Nagy [5], Adél Ádám [6], Pál Sipos [6], Eszter Farkas [7,8,9], Ferenc Bari [7] and László Janovák [1,*]

[1] Department of Physical Chemistry and Materials Science, University of Szeged, Rerrich Béla tér 1, H-6720 Szeged, Hungary; m.abdelghafour2015@yahoo.com (M.M.A.); agoston.orban.99@gmail.com (Á.O.); dagota13@yahoo.com (Á.D.)
[2] Department of Chemistry, Faculty of Science, Zagazig University, Zagazig 44519, Egypt
[3] Department of Organic and Pharmaceutical Technology, Faculty of Chemistry, Wrocław University of Science and Technology, Wybrzeże Wyspiańskiego 27, 50-370 Wrocław, Poland; lukasz.lamch@pwr.edu.pl
[4] Department of Organic Chemistry, University of Szeged, Dóm tér 8., H-6720 Szeged, Hungary; frank@chem.u-szeged.hu
[5] Department of MOL Department of Hydrocarbon and Coal Processing, Faculty of Engineering, University of Pannonia, Egyetem str. 10., H-8200 Veszprém, Hungary; nroland@almos.uni-pannon.hu
[6] Department of Inorganic and Analytical Chemistry, University of Szeged, Dóm tér 7, H-6720 Szeged, Hungary; adelada@chem.u-szeged.hu; sipos@chem.u-szeged.hu
[7] Department of Medical Physics and Informatics, Faculty of Medicine & Faculty of Science and Informatics, University of Szeged, Korányi fasor 9, H-6720 Szeged, Hungary; eszter.farkas.szeged@gmail.com (E.F.); bari.ferenc@med.u-szeged.hu (F.B.)
[8] HCEMM-USZ Cerebral Blood Flow and Metabolism Research Group, University of Szeged, Dugonics square 13. H-6720, Szeged, Hungary
[9] Department of Cell Biology and Molecular Medicine, Faculty of Science and Informatics & Faculty of Medicine, University of Szeged, Somogyi str. 4. H-6720, Szeged, Hungary
* Correspondence: janovakl@chem.u-szeged.hu; Tel.: +36-62-544-210; Fax: +36-62-544-042

1. Calculation of solubility parameters for appropriate solvents toward PES oligomers purification

The well-known methods for solubility and miscibility parameters, taking into account their chemical structure (*i.e.* number of particular chemical groups and their molar volumes), includes Hoy, Hoftyzer-Van Kreveln, Small, Hayes and Hansen. The mentioned approaches are widely used in prediction of polymers properties (e.g. solubility in various solvents, miscibility or tendency to form blends at various temperatures, wettability, internal pressure, refractive indices and dipole moment) as well as for characterization of numerous dyes, pigments, fillers or fibers in ink and coatings industries [22,23]. Generally, the mentioned methods constitute comparing of the solubility parameters' components (dispersion forces, polar forces and hydrogen bonding) or their combinations with appropriate values of critical solubility or miscibility parameters, calculated for solvents and polymers [23]. Nowadays, the mentioned approaches – even using the simplest cohesive-energy based method – were successfully exploited in the field of drug compatibility with polymer matrix [24,25]. It is worth noticing, that the mentioned strategies were used even for relatively low molecular weight copolymers (mean M_w of hydrophobic fragment 200 – 4000 Da), although they was originally designed for high-molecular weight polymers with very large number of repeating units [22-26]. Unfortunately, the mentioned theoretical estimations, based on increment group approach-

es, have to face several limitations, especially connected with no parameter representing influence of temperature (only miscibility parameter – χ – takes into consideration temperature but it is rather limited to values near room temperature due to unknown behavior e.g. near phase transition points) as well as over- or underestimation of some components, e.g. hydrogen bonding or polar forces. Therefore, use of the single calculation method may result in flawed results, but comparison of at least two or three approaches seems to be useful e.g. for studying of compatibility between the drugs and the polymer carrier matrix [24,25].

The solubility parameters for PES polymers and the solvents were calculated by group contribution methods, according to Fedors, Hoy and Hoftyzer-van Krevelen. The values of cohesive energies (Fedors's group increment method) were used for calculation of total solubility parameters (δ) of both solvents and the polymers using the same equation:

$$\delta = \sqrt{\frac{E_{coh}}{V}} \quad (1)$$

where E_{coh} denotes cohesive energy of the molecules, while V – its molar volume.

The components of dispersion forces (δ_d), polar forces (δ_p) and hydrogen bonding (δ_h) for the solubility parameters of organic solvents ($\delta^{(S)}$) and poly(ethylene succinate) oligomers ($\delta^{(P)}$) were calculated according to group contribution methods by Hoy and Hoftyzer-van Krevelen. For PES polymer calculations three different oligomers – obtained in 60 min, 70 min and 80 min synthesis (denoted as PES60, PES70 and PES80, respectively) – were chosen for calculations due to possible effect of ending groups (according to NMR measurements ending groups influence should be considered due to relatively low M_n values ranging from ca 1000 to 1300 g/mol for the mentioned polymers).

For Hoy's model the additive molar functions for the attraction functions (F_t), the polar components (F_p), the Lydersen corrections for non ideality (Δ_T) and molar volumes (V) were used for calculations (separately for the polymer and low molecular weight solvents), utilizing appropriate increment groups [23]. The appropriate auxiliary equations were used to calculate values of $\alpha^{(S)}$ (for solvents), $\alpha^{(P)}$ and n (for polymer):

$$\text{Log}\, \alpha^{(S)} = 3.39 \log(T_b/T_{cr}) - 0.1585\text{-log}V \quad (2)$$

$$T_b/T_{cr} = 0.567 + \Delta_T - (\Delta_T)^2 \quad (3)$$

$$\alpha^{(P)} = 777\, \Delta_T/V \quad (4)$$

$$n = 0.5/\Delta_T \quad (5)$$

For the low molecular weight solvents the components ($\delta_d^{(S)}$, $\delta_p^{(S)}$ and $\delta_h^{(S)}$) as well as appropriate auxiliary parameter ($\delta_t^{(S)}$) were calculated according to the following equations:

$$\delta_t^{(S)} = (F_t+277)/V \quad (6)$$

$$\delta_p^{(S)} = \delta_t^{(S)} \sqrt{\frac{1}{\alpha^{(S)}} \frac{F_p}{F_t + 277}} \quad (7)$$

$$\delta_h^{(S)} = \delta_t^{(S)} \sqrt{\frac{\alpha^{(S)} - 1}{\alpha^{(S)}}} \quad (8)$$

$$\delta_d^{(S)} = \sqrt{\left(\delta_t^{(S)}\right)^2 - \left(\delta_p^{(S)}\right)^2 - \left(\delta_h^{(S)}\right)^2} \quad (9)$$

The similar equations were utilized for calculations of solubility parameter components ($\delta_d^{(P)}$, $\delta_p^{(P)}$, $\delta_h^{(P)}$ and auxiliary $\delta_t^{(P)}$) for the polymers (PES60, PES70 and PES80).

$$\delta_t^{(P)} = (F_t + 277/n)/V \quad (10)$$

$$\delta_p^{(P)} = \delta_t^{(P)} \sqrt{\frac{1}{\alpha^{(P)}} \frac{F_p}{F_t + 277/n}} \quad (11)$$

$$\delta_h^{(P)} = \delta_t^{(P)} \sqrt{\frac{\alpha^{(P)} - 1}{\alpha^{(P)}}} \quad (12)$$

$$\delta_d^{(P)} = \sqrt{\left(\delta_t^{(P)}\right)^2 - \left(\delta_p^{(P)}\right)^2 - \left(\delta_h^{(P)}\right)^2} \quad (13)$$

The components (according to Hoftyzer-van Krevelen) of solubility parameters for both the polymer and organic solvents were calculated directly using the given equations:

$$\delta_d = \sqrt{\frac{F_d}{V}} \quad (14)$$

$$\delta_p = \frac{\sqrt{F_d^2}}{V} \quad (15)$$

$$\delta_h = \sqrt{\frac{E_h}{V}} \quad (16)$$

The solubility of the polymer in the solvent ($\Delta\delta$) is given by the equation:

$$\Delta\delta = \sqrt{\left(\delta_d^{(P)} - \delta_d^{(S)}\right)^2 + \left(\delta_p^{(P)} - \delta_p^{(S)}\right)^2 + \left(\delta_h^{(P)} - \delta_h^{(S)}\right)^2} \quad (17)$$

when particular components (dispersion forces (δ_d), polar forces (δ_p) and hydrogen bonding (δ_h)) for the solubility parameters are known (i.e. for Hoy and Hoftyzer-van Krevelen approaches).

For Fedors cohesive energy approach the solubility of the polymer in the solvent ($\Delta\delta$) is just given by the simply Euclidean distance:

$$\Delta\delta = \sqrt{\left(\delta^{(P)} - \delta^{(S)}\right)^2} \quad (18)$$

For good (thermodynamic) solubility value of Δδ should be small – in literature different cutoff values are described (e.g. 5 $MPa^{0.5}$, 7 $MPa^{0.5}$ or even 15 $MPa^{0.5}$), but 10 $MPa^{0.5}$ seems to describe boarder between solubility and insolubility [22-26]. On the other hand, due to less accuracy of Fedors's approach, the cutoff values were set as 5 $MPa^{0.5}$ [22].

The values of Δδ were compared with the limiting number (i.e. 10 $MPa^{0.5}$ or 5 $MPa^{0.5}$) and described by value 1 for thermodynamic solubility and 0 for thermodynamic insolubility. Moreover, for numerous organic solvents the particular solubility parameters (δ – total solubility parameter) and their components for dispersion forces ($δ_d$), polar forces ($δ_p$) and hydrogen bonding ($δ_h$) were assessed experimentally and taken from [22]. That is why our considerations for organic solvents include both values calculated by appropriate methods (group increments methods by Fedors, Hoy and Hoftyzer-van Krevelen) as well as experimental data from literature [22].

Table S1. Solubility of PES50, PES60 and PES70 in organic solvents according to Fedors approach (calculated and experimental values for solvents).

Solvent	calculated values for solvent			experimental values for solvent [22]		
	PES60	PES70	PES80	PES60	PES70	PES80
dioxane	0	0	0	1	1	1
chloroform	0	0	0	0	0	0
dichlorometane	0	0	0	1	1	1
hexane	0	0	0	0	0	0
ethyl acetate	0	0	0	0	0	0
THF	0	0	0	1	1	1
methanol	1	1	1	0	0	0
ethanol	1	1	1	0	0	0
acetone	0	0	0	1	1	1
DMF	1	1	1	1	1	1
toluene	0	0	0	0	0	0

Table S2. Solubility of PES50, PES60 and PES70 in organic solvents according to Hoftyzer-van Krevelen approach (calculated and experimental values for solvents).

Solvent	calculated values for solvent			experimental values for solvent [22]		
	PES60	PES70	PES80	PES60	PES70	PES80
dioxane	1	1	1	1	1	1
chloroform	0	0	0	1	1	1
dichlorometane	0	0	0	1	1	1
hexane	0	0	0	0	0	0
ethyl acetate	1	1	1	1	1	1
THF	1	1	1	1	1	1
methanol	0	0	0	0	0	0
ethanol	1	1	1	1	1	1

acetone	0	0	0	0	0	0
DMF	0	0	0	0	0	0
toluene	0	0	0	0	0	0

Table S3. Solubility of PES50, PES60 and PES70 in organic solvents according to Hoy approach (calculated and experimental values for solvents).

Solvent	calculated values for solvent			experimental values for solvent [22]		
	PES60	PES70	PES80	PES60	PES70	PES80
dioxane	1	1	1	0	0	0
chloroform	1	1	1	0	0	0
dichlorometane	1	1	1	1	1	1
hexane	0	0	0	0	0	0
ethyl acetate	1	1	1	1	1	1
THF	1	1	1	1	1	1
methanol	0	0	0	0	0	0
ethanol	0	0	0	1	1	1
acetone	1	1	1	1	1	1
DMF	1	1	1	1	1	1
toluene	1	1	1	0	0	0

For the studied systems and methods solubility / insolubility of all PES polymers (for polycondensation times equal to 60 min, 70 min and 80 min) is exactly the same for the particular organic solvent. The mentioned findings suggest that even for oligomers influence of polymer chain length and the ending groups is negligible and has no significant impact on solubility of the particular products. In order to find "good" and "bad" solvents for PES purification it is needed to prove solubility (for "good" solvent) or insolubility (for "bad" solvent) by agreement between different approaches. In general solubility of PES oligomers was confirmed by all methods and approaches (excluding highly inaccurate Fedors's approach with calculated values of solubility parameters for solvents) for dioxane (1,4-dioxane) and THF, while their insolubility – for hexane and methanol. Taking into consideration the mentioned solvents' properties – especially their miscibility with water (crude oligomers contain considerable amounts of water) and boiling points (in order to enable fast and complete evaporation of a solvent after purification) – the best PES purification method should be precipitation from THF ("good" solvent) with methanol ("bad" solvent). Both solvents are freely miscible with water as well as are characterized by low boiling points (below 70 °C), in contrast to hexane (water immiscible compound) and 1,4-dioxane (boiling point 101 °C). Confirmation of thermodynamical miscibility / immiscibility of PES polymer in the mentioned organic solvents (THF and methanol) indicates optimal conditions for purification process in order to avoid unnecessary losses and possible repeated purification for different solvent mixtures.

2. Molecular weight determination

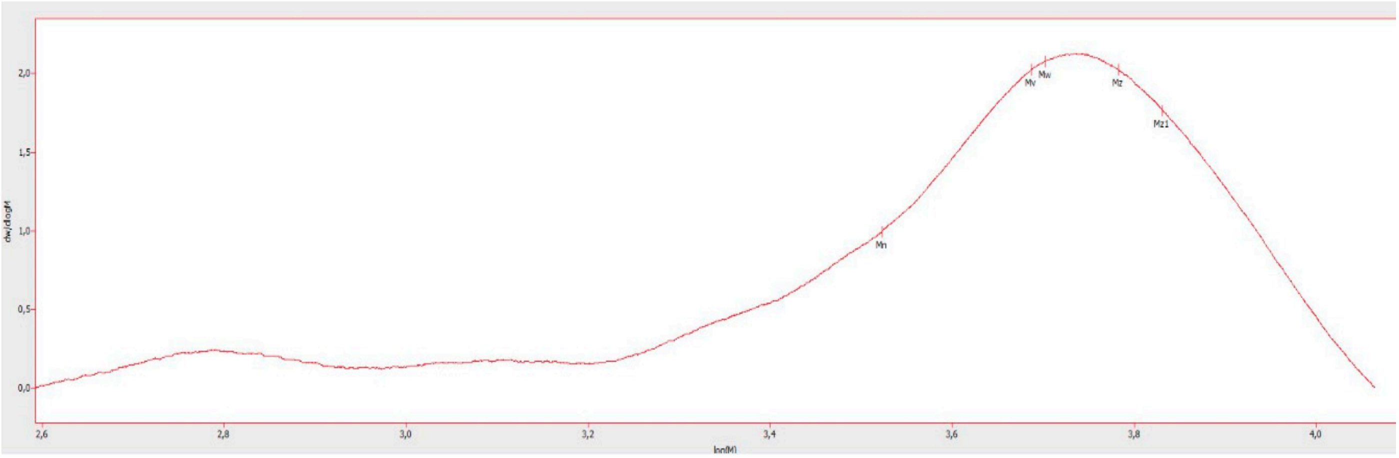

Figure S1. GPC molecular weight distribution curve obtained for polyester with 80 min polycondensation time

To back up the results of the NMR measurements, the average molecular weight of the PES products was also measured with mass spectroscopy (MS). A typical MS spectrum of them is presented on **Figure S2.**

Studying the spectra the typical masses of deprotonated PES with the supposed structure can be found (*e.g.* at 1025, 881 or 737 m/z). The results also suggest that there are species present which do not contain the ethylene glycol end (end group II on **Figure 4.**) but have another carboxylic end group. The one and two-fold deprotonated masses of such structures can also be observed on the spectra. Average molecular weight of the PES samples were calculated according to these spectra considering the relative abundance of the different species.

$M_n = \Sigma_i (\text{relative abundace}_i * M_i) / \Sigma_i \text{ relative abundance}_i$

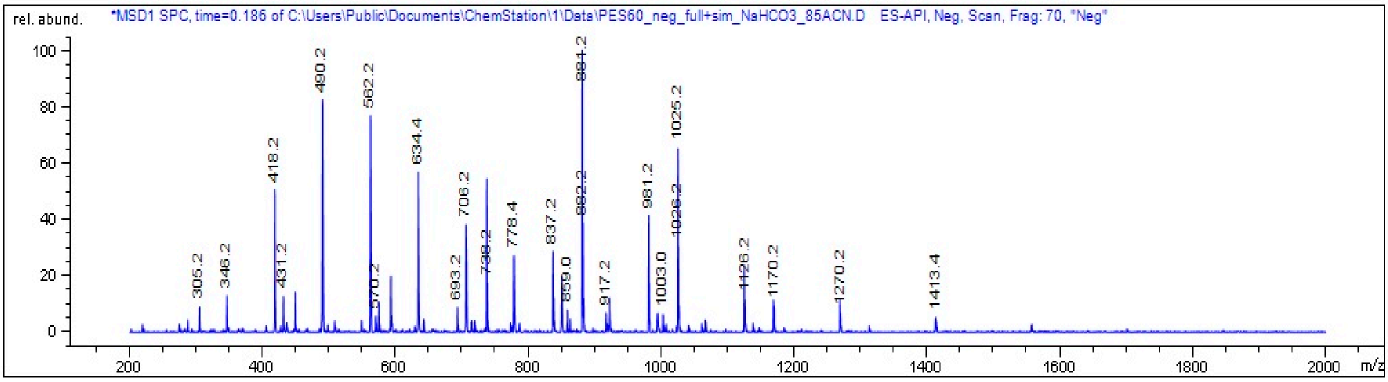

Figure S2. Representative MS-spectrum of the measured PES sample (80 min polycondensation time)

3. Estimation of the volume fraction of water in DMSO:water mixtures for PES precipitation – the theoretical approach

The calculation of appropriate critical values of Flory-Huggins interaction parameter (χ_{crit}) and polymer volume fraction (φ_{crit}) enables studying polymer solubility in mixtures of solvents. Precipitation of poly(ethylene succinate) from DMSO:water mixtures may constitute crucial step of different PES-based nanocarriers for biologically active compounds. That is why the mentioned behavior was carefully studied by means of turbidimetry (see Figure 6 and 7 in the main article). In order to gain additional information about the studied systems (PES in DMSO:water mixtures) appropriate calculations were performed. It is not possible to find appropriate group increments for calculation of total solubility parameter and its particular components (dispersion forces (δ_d), polar forces (δ_p) and hydrogen bonding (δ_h)) for water and DMSO so the mentioned values were taken from [22]. Taking into account the limited accuracy of the mentioned approach (critical values constitute estimates based on only limited experimental data) only Hoy's method was used for polymer solubility parameter and its components. Moreover, polymer was approximated by taking into account only the repeating unit, comprising two methylene and one ester group. The obtained results were showed graphically using two-dimensional plots (δ_a versus δ_V) by introducing appropriate auxiliary parameters for both the polymer and solvents:

$$\delta_a = \sqrt{(\delta_p)^2 + (\delta_h)^2} \quad (19)$$

$$\delta_V = \sqrt{(\delta_d)^2 + (\delta_p)^2} \quad (20)$$

The center of the circle, marked with "x" symbol, denotes parameters δ_a and δ_V for PES (16.7 MPa$^{0.5}$ and 19.8 MPa$^{0.5}$), while its radius (the radius of interaction) depends on the molecular volumes of water and DMSO (values taken from [22]). Datapoints for water and DMSO mark coordinates of δ_a and δ_V values and are colored in agreement with appropriate circles, red for DMSO and black for water, respectively (see Figure S1). Generally, if the point is situated inside appropriate (i.e. of the same color) circle PES should be soluble in the mentioned solvent.

The calculated values of the solubility parameters of the polymer and solvents were used to calculate the miscibility parameter (Flory-Huggins interaction parameter – χ) for DMSO and water:

$$\chi = \frac{V}{RT}\left(\delta^{(P)} - \delta^{(S)}\right)^2 \quad (21)$$

V denotes the molar volume of the studied drug, R – universal gas constant, while T is the standard temperature in Kelvins (here 298 K). The mentioned values were found to be equal to 0.52 and 4.69 for DMSO and water, respectively.

The critical values of Flory-Huggins interaction parameter (χ_{crit}) and polymer volume fraction (φ_{crit}) were estimated for the given polymerization degree (x = 12.67, maximal value of polymerization degree for PES 80 min, corresponding with the less favorable conditions for polymer solubility – the polymers characterized by lower values of polymerization degree will be better soluble in each systems, according to the theory) of poly(ethylene succinate):

$$\chi_{crit} = \frac{1}{2} + \frac{1}{\sqrt{x}} + \frac{1}{2x} \quad (22)$$

$$\varphi_{crit} = \frac{1}{1+\sqrt{x}} \quad (23)$$

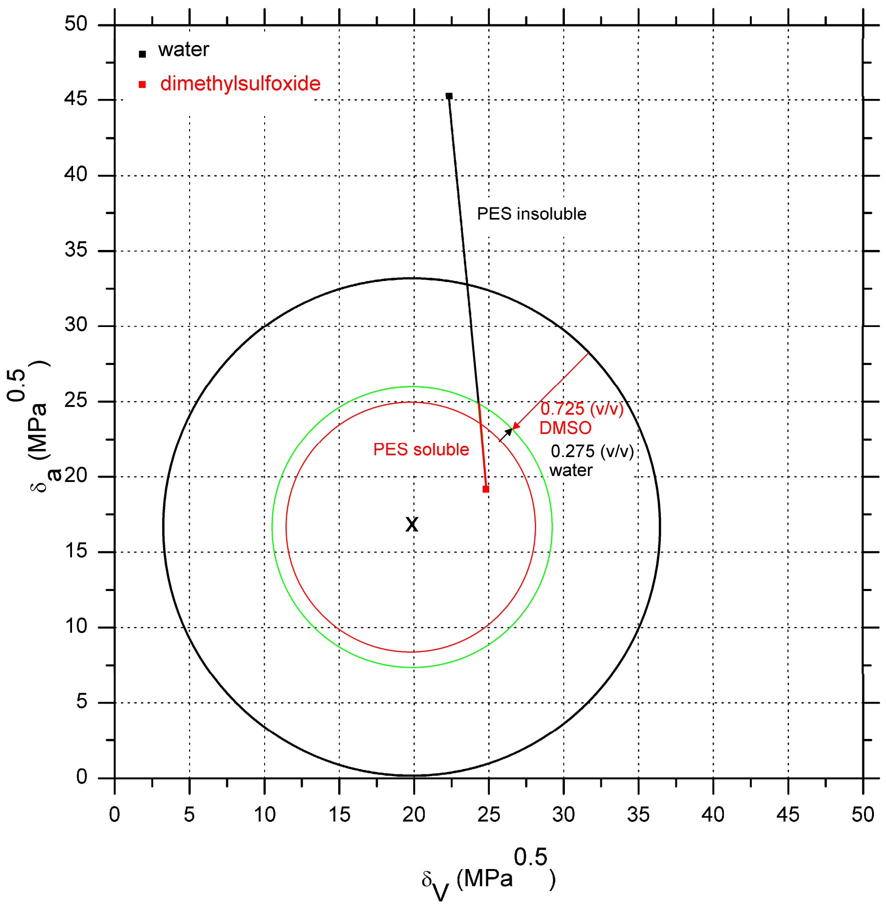

Figure S3. Solubility of PES in water, DMSO and their mixtures (green circle) – determination of minimal water fraction in DMSO:water system when precipitation of the polymer may be observed.

Solubility parameters and their components for mixtures of solvents may be calculated as volume weighted average for the composition. This property enables studying the composition of solvent and non-solvent in which the polymer is thermodynamically insoluble, so the precipitation should appear. Solubility of poly(ethylene succinate) in DMSO / water mixtures was studies and it was found that the polymer should precipitate when volume fraction of water exceeds 0.275. These findings were confirmed experimentally – turbidity of PES solution in DMSO started to appear when around 0.3 (v/v) of water was added, especially for polymers with lower molecular weight. For PES with higher value of molecular weight the precipitate appears when amount of the added water exceeds around 0.4 (v/v), most possibly due to kinetic reasons (slower conformation changes for larger macromolecules), although the mentioned effect may be more complicated for oligomers (considerable influence of ending groups).

The critical values of Flory-Huggins interaction parameter (χ_{crit}) and polymer volume fraction (φ_{crit}), estimated for of PES (polymerization degree 12.67), were found to be equal to 0.82 and 0.22, respectively. The mentioned parameters are in good agreement with Flory-Huggins interaction parameters, calculated for PES in DMSO and water – for DMSO, where PES was found to be soluble in it, the value of χ is lower than χ_{crit} i.e. 0.82. Moreover, the used solutions of PES in DMSO (concentration of 6 %) were found to be stable according to the calculations (volume fraction of polymer does not exceed 0.22).
